# Towards malaria elimination: analysis of travel history and case forecasting using the SARIMA model in Limpopo Province

**DOI:** 10.1007/s00436-023-07870-y

**Published:** 2023-06-13

**Authors:** Olukunle O. Oyegoke, Taiye S. Adewumi, Samuel A. Aderoju, Ntimbane Tsundzukani, Eric Mabunda, Matthew A. Adeleke, Rajendra Maharaj, Moses Okpeku

**Affiliations:** 1 Discipline of Genetics, School of Life Sciences, University of KwaZulu-Natal, Durban, South Africa; 2 Department of Mathematics and Statistics, Kwara State University, Ilorin, Nigeria; 3 Limpopo Department of Health, Malaria Control Program, Limpopo, South Africa; 4 Malaria Research Unit, South African Medical Research Council, Durban, South Africa

**Keywords:** Imported malaria, Surveillance, Travel history, South Africa

## Abstract

Despite various efforts and policy implementation aimed at controlling and eliminating malaria, imported malaria remains a major factor posing challenges in places that have made progress in malaria elimination. The persistence of malaria in Limpopo Province has largely been attributed to imported cases, thus reducing the pace of achieving the malaria-free target by 2025. Data from the Limpopo Malaria Surveillance Database System (2010–2020) was analyzed, and a seasonal auto-regressive integrated moving average (SARIMA) model was developed to forecast malaria incidence based on the incidence data’s temporal autocorrelation. The study found that out of 57,288 people that were tested, 51,819 (90.5%) cases were local while 5469 (9.5%) cases were imported. Mozambique (44.9%), Zimbabwe (35.7%), and Ethiopia (8.5%) were the highest contributors of imported cases. The month of January recorded the highest incidence of cases while the least was in August. Analysis of the yearly figures showed an increasing trend and seasonal variation of recorded malaria cases. The SARIMA (3,1,1) X (3,1,0) [12] model used in predicting expected malaria case incidences for three consecutive years showed a decline in malaria incidences. The study demonstrated that imported malaria accounted for 9.5% of all cases. There is a need to re-focus on health education campaigns on malaria prevention methods and strengthening of indoor residual spray programs. Bodies collaborating toward malaria elimination in the Southern Africa region need to ensure a practical delivery of the objectives.

## Introduction 

The drive to reduce the malaria burden witnessed great momentum in many of the affected countries as a result of the significant inputs from the leadership in the form of political and financial commitments. One of the outcomes is the fact that 26 new countries reported a decline in local malaria cases between 2000 and 2020, and this, in some instances, has resulted in malaria elimination (WHO 2021).

In spite of these various efforts and policies implementation, imported and cross-border malaria remain major factors posing great challenges (either as re-introduction or mitigation of elimination) in places that have made significant progress in malaria elimination (Lehky Hagen et al. 2005; Wangdi et al. 2015). According to MESA (2022), “border malaria is defined as the malaria transmission or potential for transmission that takes place across or along borders between countries sharing a land border and is frequently cited as a challenge to malaria elimination,” while World Health Organization (2018) described an imported case as “one that is due to mosquito-borne transmission which is acquired outside the area in which it was detected.” Parasite carriage through human migration often occurs from highly endemic to areas where the infection rate is low or controlled (Monge-Maillo & López-Vélez 2012), and in some cases, the issue of border sharing becomes the main factor aiding the transmission (Sturrock et al. 2015).

South Africa has three provinces (KwaZulu Natal, Mpumalanga, and Limpopo) which are still affected by malaria; however, these provinces are noted to be in different phases of malaria control and elimination (South Africa National Department of Health 2019). Migration has been reported as one of the hurdles to achieving elimination in these provinces, as particularly recently reported in the KwaZulu Natal province which records few local cases but a high number of imported cases (Raman et al. 2020). The issue of border sharing with countries such as Mozambique and Zimbabwe where malaria cases are still high has been reported to as a contributor to the persistence of malaria in parts of Limpopo (Khosa et al. 2013).

Although governmental efforts in the form of various supports and commitments to eliminating malaria in the country have been commendable, the high number of malaria cases still recorded in Limpopo Province is still a challenge. For instance, in 2017, the spontaneous surge in recorded cases had the worst toll on Limpopo Province with fifty-four malaria deaths (Ravhuhali et al. 2017) . In addition, the recent outbreak of the SARS-CoV-2–19 virus disease (COVID-19) has worsened the situation in some communities. This presented in the form of increased morbidity as a result of misdiagnosing malaria as COVID-19 because of the symptom’s similarities of both diseases at the time of presentation which ultimately resulted in 63,000 deaths worldwide (NICD 2022; World Health Organization 2022).

Anecdotal reports had it that imported malaria is the bane of malaria elimination in Limpopo Province; however, the understanding and appropriate interpretation of available data will provide an objective standpoint to this, and it will equally inform on appropriate preparedness against future malaria outbreaks. In this chapter, we used surveillance data from Limpopo Province (2010–2020) to identify the contribution of imported malaria to the persistence of malaria infection in the province based on time series data and also modeled the expected malaria incidence pattern over a 3-year period by application of seasonal autoregressive integrated moving average (SARIMA).

## Methodology

Malaria is a disease that requires notification in South Africa. Over the years, the means of notification have been paper-based, and until recently, there is gradual migration to a computer-based system. Hence, the current practice of data collection is a mixture of both systems. Secondary data retrieved from the Limpopo Malaria Surveillance Database System were used for this study.

### Study location

**Fig. 1:**
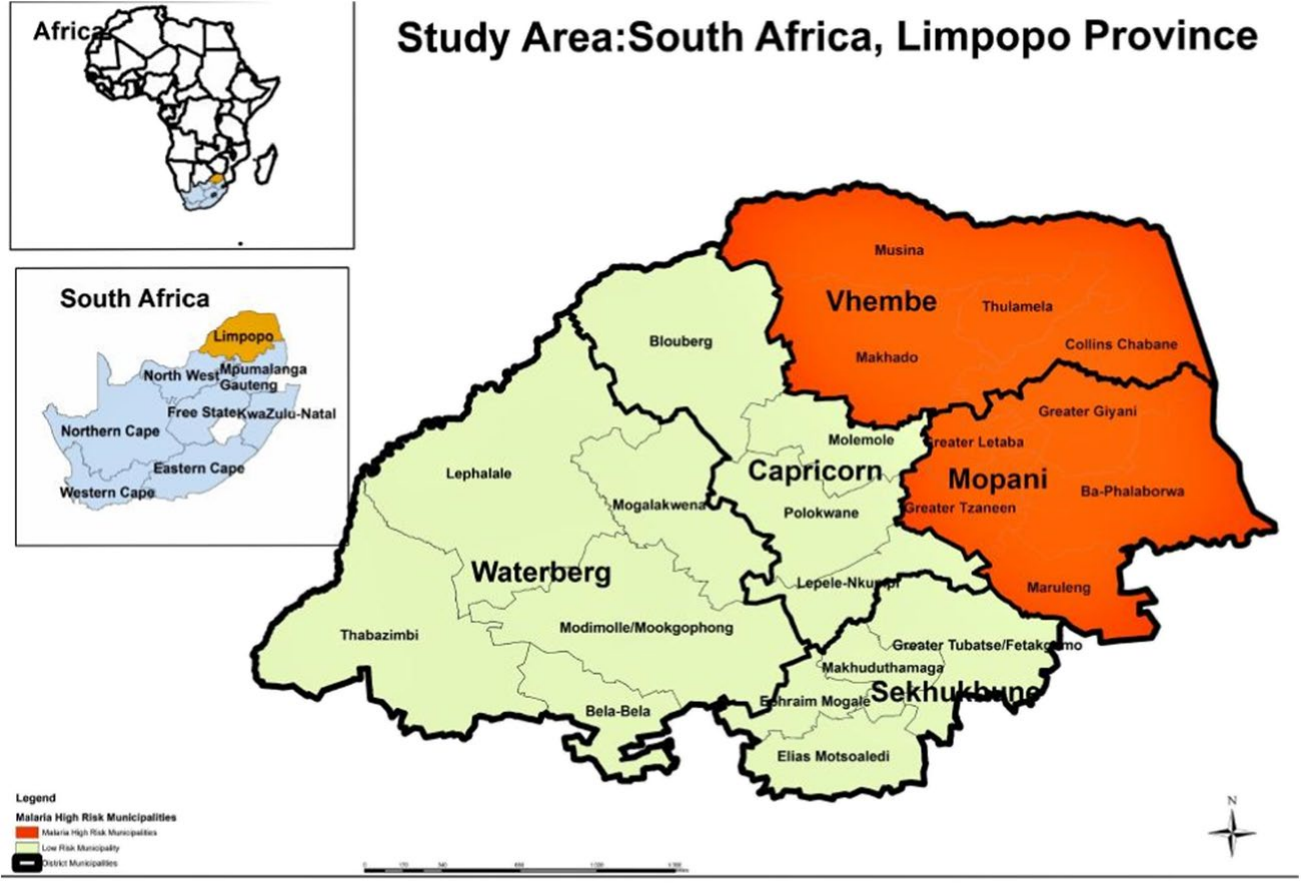
Map of South Africa with the districts in Limpopo Province showing degrees of malaria-endemic regions

Limpopo Province is located in the north-east part of South Africa at coordinates 22–25° S, 27–32° E with a population estimate of 5.4 million (Stats SA 2022) (Fig. 1). It has five districts that are mostly rural: Waterberg, Capricorn, Vhembe, Greater Sekhukhune, and Mopani. The province is situated in the part of the country that is known for the hot weather conditions in the summer and is prone to extreme drought due to unpredictable rainfall and severe water shortage (Sikhwari et al. 2022). August is the month with the least month of rainfall (0.0 mm) while the highest rainfall occurs in December. The least average humidity of 48% is usually recorded in September, and the highest average of 65% is experienced in February (World Data Info 2022). Malaria is still endemic in the province; hence, cases are still recorded in all the months of the year but are more predominant between September and May (Tshiala et al. 2011; Adeola et al. 2019). The province shares a border with Zimbabwe and Mozambique which are malaria-endemic countries, and a study in Mutale municipality of Limpopo reported a high number of imported cases with the majority coming from Zimbabwe (Khosa et al. 2013).

### Surveillance data description

Analyses of the data were done for malaria seasons covering a period ranging from 2010 to 2021. These included both the recorded passive cases identified at the health facilities and the active cases identified through the case tracers. Information on the retrieved data was made anonymous except for the following: demographic details such as age and gender, locations used for data collection, diagnostic methods, and travel destinations that were located outside of South Africa.

All the identified cases based on either active or passive cases were divided into either imported (positive travel history) or local (no travel history). A malaria-positive case or infection was defined as cases that were identified using rapid diagnostic test (RDT) kits, with or without confirmation of the presence of Plasmodium by microscopy. An imported case or infection is the one obtained outside the region in which it was diagnosed, whereas a local case is one without any prior travel history but was contracted locally (World Health Organization, 2018). The testing modality was either with the use of RDT (with or without microscopy), as stipulated in the South African national guideline. Included in the study were all malaria cases confirmed by RDT and/or microscopy from the period 2010–2020. Duplicate cases were excluded from the data before statistical analysis.

### Statistical analysis

The descriptive and inferential statistical analyses were used for comparing local and imported malaria cases (2010–2020) as well as the travel patterns of the imported malaria cases. Statistical analyses were performed using Stata 14 (StataCorp 2015) and the R package (R Core Team 2022). Using the temporal autocorrelation found in the incidence data, we created a Seasonal Autoregressive Integrated Moving Average (SARIMA) model to predict malaria incidence. SARIMA models have been documented to be good for the epidemiological survey; it clearly assumes temporal dependence between observations. Its application has also been noted in describing the temporal dependence structure of a time series such as obtainable in clinical cases in which the pattern of occurrences is noted to be seasonal (Kumar et al. 2014; Helfenstein 1991; Gondwe et al. 2021). Among various disease conditions that demonstrate transmissibility and seasonality, the use of the SARIMA model is preferred due to its stronger predictive power (Anokye et al. 2018; Luz et al. 2008; Ture & Kurt 2006; Nobre et al. 2001; Gondwe et al. 2021; Liu et al. 2023). Unlike complex models which require extensive and detailed data, the stability and simplicity of SARIMA make it easily applicable in an environment with scarce resources and to deal with seasonal effects (Liu et al. 2023). Thus, making it more beneficial compared with other models (Anokye et al. 2018; Pascual et al. 2008). Pearson’s chi-square statistic was used to examine the relationship/independence of some selected categorical variables on the locations of malaria patients.

The Akaike information criterion (AIC) and Bayesian information criterion (BIC) were used to compare models of varying orders that had been fitted using R software (R Core Team 2022).

Since it is important that the model residual do not contain temporal autocorrelation, a confirmation of this was carried out using the Ljung-Box method (Burns 2002). A 36-month advance forecast was created using the selected model for the period from January 2021 to December 2023. Finding out which model performs better for immediate, short-term goals as opposed to longer-term (annually) forecasting of future malaria patterns was the main goal. We calculated the mean absolute percentage error (MAPE), root mean squared error (RMSE), mean absolute error (MAE), and mean absolute scaled error (MASE) among others to compare the accuracy of out-of-sample forecasts across different models as suggested by the ACF and PACF. As a sequel to this, model forecasts along with 95% prediction intervals were plotted.

## Results

### Findings from the general survey

All data received from the different locations covering 132 months (January 2010 to December 2020) were a total of 60,244 case observations. These were analyzed, and categorization was made as a local or an imported case (Table 1). Of the 57,292 people that were tested, 51,823 (90.5%) cases were local while 5469 (9.5%) cases were imported. More males 29,117 (56.2%) and 4221 (77.3%) were infected over the years among the local and imported cases categories respectively (*X*^2^ = 896.363, *P* < 0.001). Case fatality showed that 545 (1.1%) deaths were recorded among the local cases for the years evaluated, while 68 (1.2%) were deaths among the imported malaria group (*X*^2^ = 1.718, *P* = 0.190).

**Table 1:** Cross-tabulation of some selected variables versus the location of malaria patients

Variables	Categories	Malaria local or imported		Chi-squares, X^2^ (*P*-value)
		Local (count/percentage)	Imported (count/percentage)	
Malaria death (*N* = 57,292)	No	51278 (98.9%)	5401 (98.8%)	1.718 (0.190)
	Yes	545 (1.1%)	68 (1.2%)	
Symptomatic (*N* = 10,622)	No	116 (1.2%)	9 (1.5%)	0.454 (0.561)
	Yes	9890 (98.8%)	607 (98.5%)	
Previous episode of malaria illness (*N* = 38,823)	No	35370 (97.9%)	2648 (98.7%)	9.213 (0.002)
	Yes	771 (2.1%)	34 (1.3%)	
House sprays (*N* = 37,659)	No	13389 (38.0%)	1981 (82.2%)	2563.481 (< 0.001)
	Yes	5563 (15.8%)	52 (2.2%)	
	Unknown	16298(46.2%)	376 (15.6%)	
Protection against malaria (*N* = 38,824)	No	35210 (97.4%)	2661 (99.1%)	29.347 (< 0.001)
	Yes	929 (2.6%)	24 (0.9%)	
First malaria episode (*N* = 57,292)	No	16101 (31.1%)	2807 (51.3%)	918.026 (< 0.001)
	Yes	35722 (68.9%)	2662 (48.7%)	
Sex (*N* = 57,289)	Female	22703 (39.6%)	1248 (22.8%)	896.363 (< 0.001)
	Male	29117 (56.2%)	4221 (77.3%)	
Age group (*N* = 57,288)	< 10 years	10655 (20.6%)	479 (8.8%)	1971.122 (< 0.001)
	10 – 19 years	11801 (22.8%)	497 (9.1%)	
	20—29 years	9686 (18.7%)	1807 (33.0%)	
	30—39 years	7381 (14.2%)	1413 (25.8%)	
	40—49 years	5156 (10.0%)	808 (14.8%)	
	50—59 years	3664 (7.1%)	313 (5.7%)	
	> 59 years	3476 (6.7%)	152 (2.8%)	

Among the local cases, the highest number of malaria incidents occurred in the 10–19 years age category which recorded 11,801 (22.8%), followed by children less than 10 years—10,655 (20.6%); on the other hand, the age categories 20–29 years recorded the highest number of imported cases with 33.0% (*X*^2^ = 1971.122, *P* < 0.001). Out of those that reported a history of previous malaria infection, there were 771 (2.1%) people that were local cases and had at least one episode of malaria in the past, but only 34 (1.3%) were reported among the imported cases (*X*^2^ = 9.213, *P* = 0.002). A large proportion of both the local 35,210 (97.4%) and imported cases 2661 (99.1%) reported non-use of any form of malaria protective measures compared with the number that used protective measures (*X*^2^ = 29.347 *P* < 0.001). Similarly, it was observed from the data that a greater proportion of both local 13,389 (38.0%) and imported cases in 1981 (82.2%) did not report having any form of house spray (*X*^2^ = 2563.481, *P* < 0.001). Although > 40% did not indicate the status of house spray among the local respondents, only 52 (2.2%) imported cases reported benefiting from house spraying.

### Analysis of reported malaria cases by locations

**Fig. 2:**
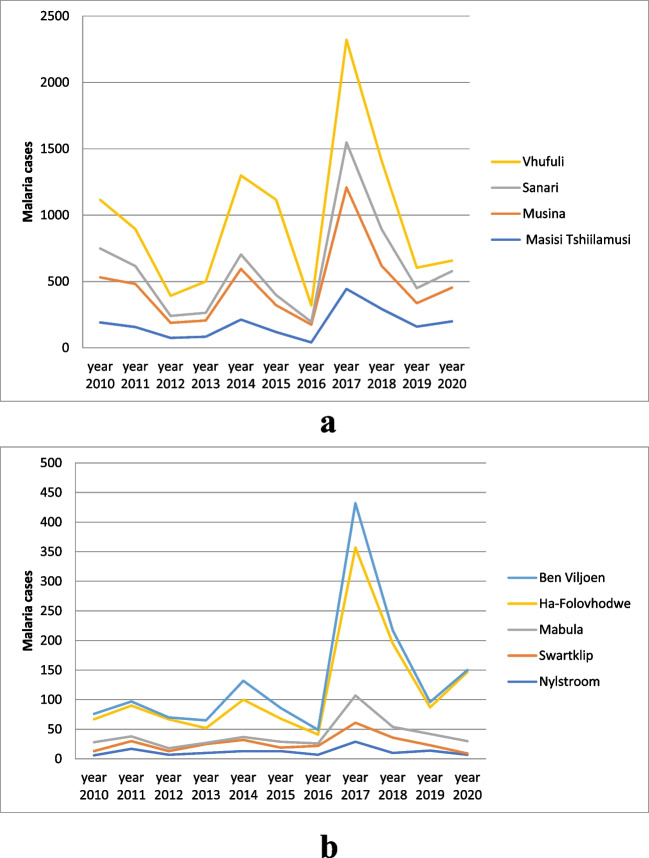
**a** Graph showing malaria incidences in locations with a high number of cases. **b** Graph showing malaria incidences in locations with a low number of cases

Due to inconsistency in the report from some of the locations, we analyzed the data from places that had full data (17% of total data collection sites) and noted that the total number of malaria cases recorded was 19,003 out of which 14,097 (74.2%) accounted for local cases (no travel history) and 4906 (25.8%) were imported (positive travel history). Although these sites are located in different parts of the province, sites that recorded high numbers of malaria cases are located close to the border. The malaria incident pattern was fluctuating from 2010 to 2015 with the least number of cases recorded in 2016 (586 cases) followed by a notable case outbreak in 2017 (4822 cases), and it has been on a downward trend since 2018. Vhufuli, Musina, Sanari, and Masisi recorded the highest number of malaria cases from 2010 to 2020, with the following as accumulated total for the evaluated period: 3993, 3140, 1977, and 1521, respectively (Fig. 2a). Altogether, these four locations accounted for 56% (10,631/19,003) of the total cases. The locations with low incidences (< 200 cases) of malaria include over the 11-year period are Nylstroom (133), Swartklip (150), Mabula (153), Ha-Folovhodwe (177), and Ben Viljoen (199), thereby comprising 4.3% (812/19,003) of the total cases (Fig. 2b). The majority of the imported cases were found in Musina—32.4% (1591/4906) followed by Vhufuli—9% (441/4,906), Polokwane—6.6% (326/4906), and Bochum—6% (295/4906). Travel history was least reported in Tshidimbini, Tshitavha, and Mooihoek.

### Positive travel history by source country

The country of departure is reported by those with imported cases, the majority of which are Africans, and these have been grouped according to their different continental region as indicated in Table 2. They are the Southern Africa region (Mozambique, Zimbabwe, Botswana, Lesotho, Namibia, Malawi, Swatini, Zambia), the East Africa region (Angola, Ethiopia, Kenya, Somalia, South Sudan, Tanzania, Uganda), the West Africa region (Nigeria, Ghana, Sierra Leone), and the Central Africa region (Democratic Republic of Congo, Equatorial Guinea, Cameroun). Three countries were outside Africa—Afghanistan, Bangladesh, and Pakistan. The main contributors of imported cases were Mozambique with 2370 (44.9%), Zimbabwe with 1882 (35.7%), and Ethiopia with 446 (8.5%). The year 2017 witnessed the highest number of imported cases with a recorded of 776 imported cases. Non-African countries that contributed to the imported cases over the 11-year period were Pakistan, Bangladesh, and Afghanistan with 5, 1, and 1 cases, respectively (Table 2).

**Table 2:** Travel source of imported malaria infection

Countries	Imported malaria cases	Percent
Mozambique	2394	43.8
Zimbabwe	1889	34.5
Ethiopia	447	8.2
Somalia	192	3.5
South Africa	164	3.0
Malawi	125	2.3
Zambia	72	1.3
Congo (DRC)	54	1.0
Botswana	33	0.6
Pakistan	18	0.3
Uganda	14	0.3
Angola	13	0.2
Ghana	9	0.2
Kenya	9	0.2
Eswatini	7	0.1
Nigeria	7	0.1
Sierra Leone	6	0.1
Cameroon	5	0.1
Namibia	4	0.1
Rwanda	4	0.1
Tanzania	2	0.04
Equatorial Guinea	1	0.02
Lesotho	1	0.02

### Trend analysis and model forecast of anticipated malaria cases

**Fig. 3:**
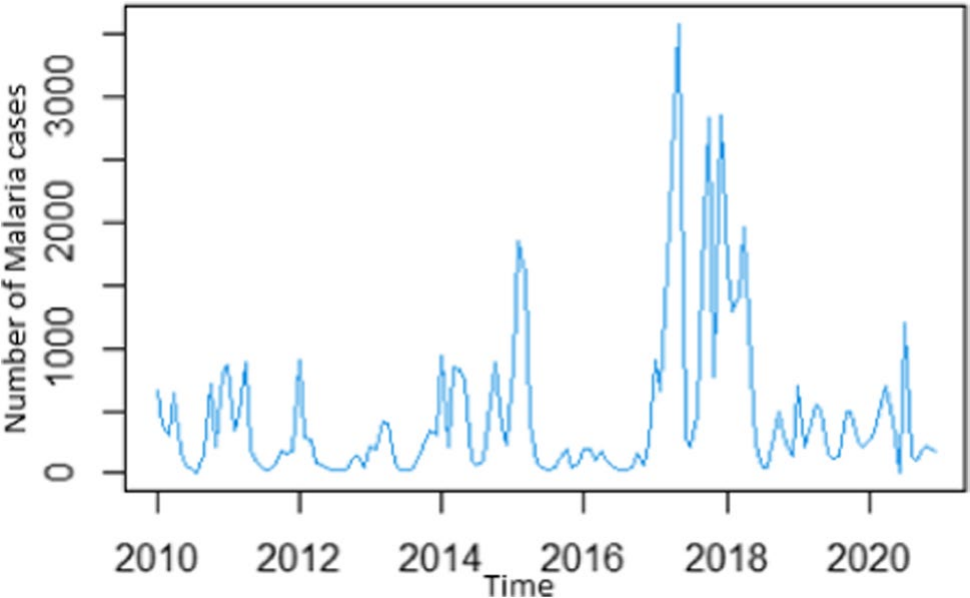
Time Plot of the cases of Malaria from January 2010 to December 2020

**Fig. 4:**
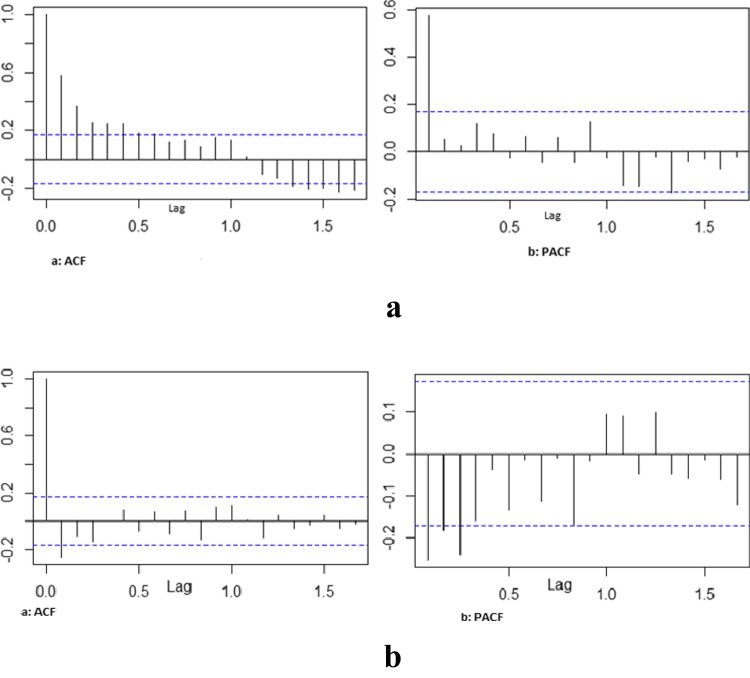
**a** The generated autocorrelation function (ACF) and partial autocorrelation function (PACF) graphs of the observed data. **b** The generated autocorrelation function (ACF) and partial autocorrelation function (PACF) graphs of the differenced data

The spectrum of data collected from January 2010 to December 2020 affords the opportunity to do a time series analysis on the malaria case incidences in Limpopo Province. The first step was the plotting of malaria cases against time to detect and correct the form of non-stationarity of the time series (Fig. 3) and identified autoregressive and moving average terms needed by calculating the autocorrelation function (ACF) and partial autocorrelation (PACF) functions (Fig. 4). The result of the Dickey-Fuller test of the stationarity of the data was − 2.1496 (*p*-value = 0.5143), which revealed that the data were not stationary. However, after the first differencing, the *p*-value of 0.0183 (less than 0.05) indicated that the data was stationary after first differencing.

**Fig. 5:**
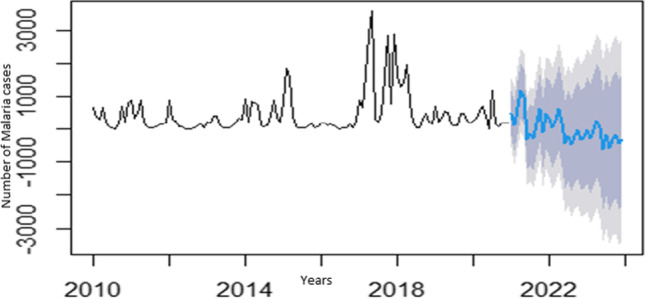
Graph depicting the observed and forecasted values for years 2021 to 2023

The outcomes showed that the SARIMA (3,1,1) x (1,1,0) was the best fit for predicting the monthly incidence of malaria for the consecutive years 2021–2023. This can be noted in the generated values of MAPE, RMSE, MAE, and MASE values as shown in Tables 3 and 4. Note that only the top model chosen was reported and used to predict the monthly incidence of malaria (Fig. 5).

**Table 3:** Accuracy test of some selected SARIMA models

Models	Accuracy measure						
	RMSE	MAE	MPE	MAPE	MASE	AIC	BIC
SARIMA (0,1,1) x (0,1,0)	0.9048	0.6506	− 1.0907	12.8222	0.6171	330.48	336.04
SARIMA (1,1,0) x (0,1,0)	0.9305	0.6789	− 1.0532	13.4874	0.6440	337.01	342.57
SARIMA (1,1,1) x (0,1,0)	0.9037	0.6508	− 1.0994	12.8235	0.6173	332.18	340.52
SARIMA (1,1,1) x (1,1,0)	0.8410	0.5804	− 1.2770	11.6030	0.5506	319.08	330.20
SARIMA (1,1,2) x (0,1,0)	0.9033	0.6506	− 1.1008	12.8230	0.6172	334.07	345.18
SARIMA (1,1,2) x (1,1,0)	0.8402	0.5803	− 1.2835	11.6060	0.5505	320.86	334.76
SARIMA (2,1,1) x (1,1,0)	0.8365	0.5764	− 1.2939	11.5532	0.5467	319.92	333.82
SARIMA (3,1,1)x(1,1,0)	0.7529	0.5270	− 1.4940	10.7055	0.4999	307.27	329.50

**Table 4:** SARIMA (3,1, 1) x (3,1,0) [12]—coefficient and standard error of generated parameters

	Coefficient	Standard error
Non-seasonal AR(1)	− 0.4559	0.2867
Non-seasonal AR(2)	− 0.3332	0.1641
Non-seasonal AR(3)	− 0.2714	0.1160
Non-seasonal MA(1)	− 0.1026	0.2959
Seasonal AR(1)	− 0.6926	0.1197
Seasonal AR(2)	− 0.5147	0.1311
Seasonal AR(3)	− 0.1279	0.1225

**Fig. 6:**
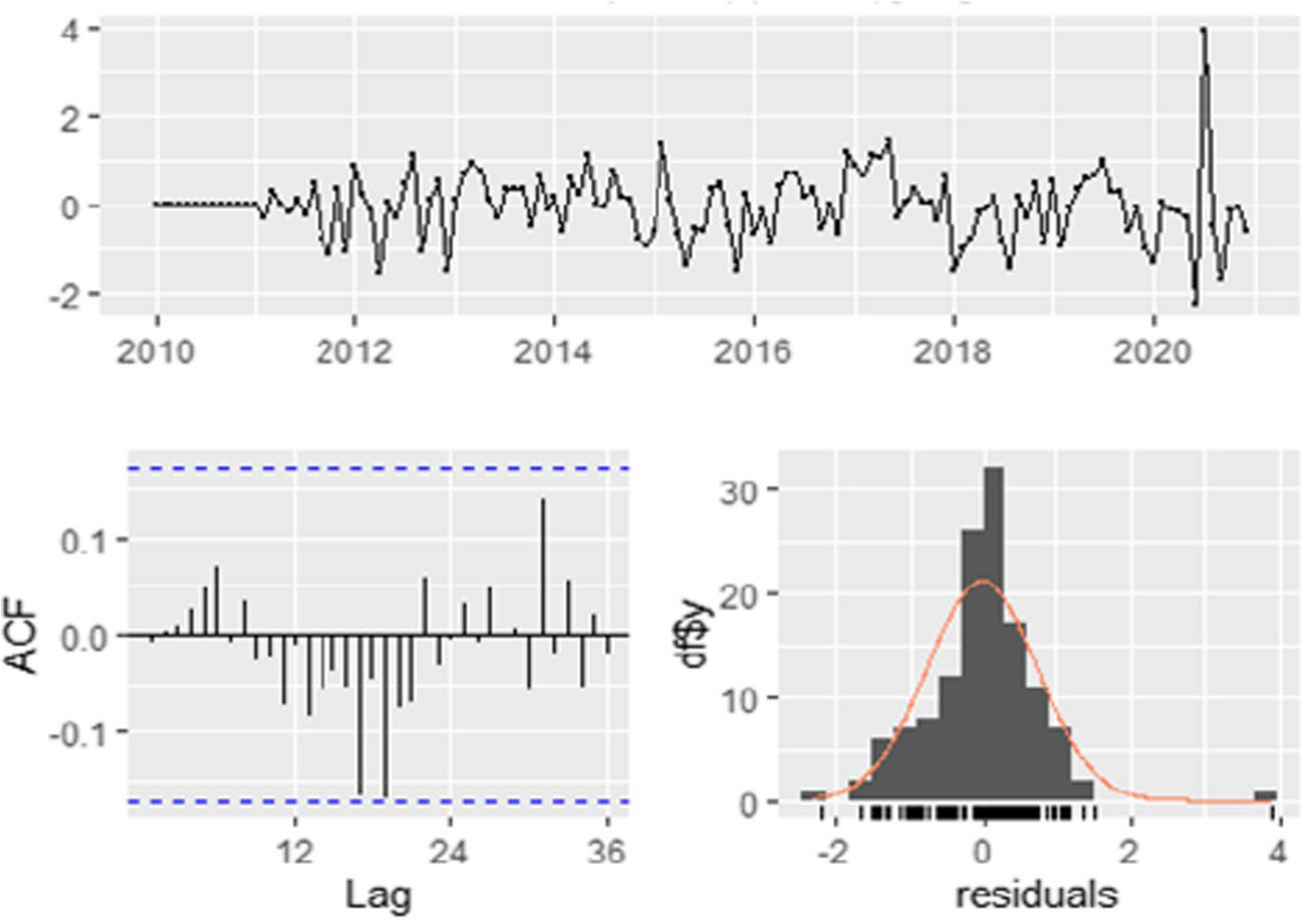
Graph showing the residuals analysis from SARIMA (3,1,1) x (3,1,0) [12]

A test of comparisons with other models indicated that SARIMA (3,1, 1) x (3,1,0) [12] was the best option. Plots of the residuals in Fig. 6 speak to this. Furthermore, it would be observed that a normal distribution was demonstrated by the histogram plot of the residuals (Fig. 6).

A portmanteau test (Ljung-Box test) returns a large *p*-value (0.5345) greater than the level of significance (0.05), also suggesting that the residuals are white noise. This implies that this model was appropriate.

**Fig. 7:**
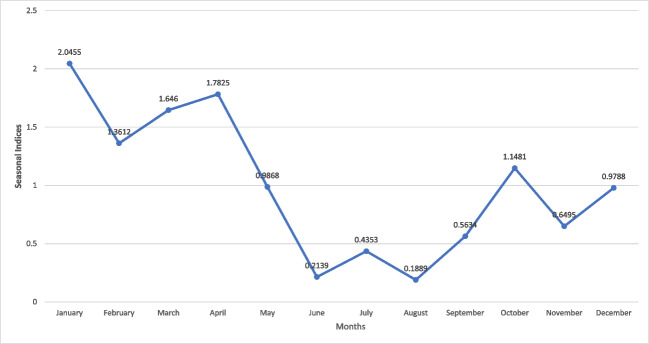
Seasonal indices graph (January 2010–December 2020)

The seasonal indices (S.I) were obtained, and this has been presented in Fig. 7. Obviously, malaria infection occurs throughout the year; however, the highest recorded incidence was in January. The other months with a high number of cases include April, March, February, and October. The risk was lowest in June, August, and July. Note that in July, the minimum and maximum reported cases from 2010 to 2019 were 20 and 217, respectively, while 1193 cases were reported in 2020.

## Discussion

South Africa projected the malaria elimination goal for the year 2025; however, there are multiple potential hurdles to be crossed for this to become a reality, and one of them is the persistence of malaria cases in the Limpopo Province. Our findings in this study showed that 9.5% (5469/57,288) of all the malaria cases diagnosed had a positive travel history outside of South Africa. It means that, in addition to other factors, imported cases are important contributors to the causes of ongoing malaria transmission in the province. The study by Khosa et al. (2013) found the imported case to be 6.6% in the Mutale municipality of Limpopo. Although this was less than the finding in this provincial study, it corroborates the persistently high number of imported cases.

Our study also observed that most of those with imported cases have a history of primary travel source being from Mozambique (44.9%) or Zimbabwe (35.7%), while a total of 16.9% had the primary travel source from Ethiopia (8.5%), Somalia (3.6%), Malawi (2.4%), Zambia (1.4%), or Congo, DRC (1%)—Table 2. In fact, the municipality study by Khosa et al. (2013) reported that Zimbabwe constituted 96.5% of all the recorded imported cases. Mozambique and Zimbabwe are sub-Saharan African countries that share a border with Limpopo Province, and they are endemic for malaria with incidence rates of 320.2 and 98.5 per 1000 population respectively (World Data Info 2022). It has been established that in settings that are nearing elimination such as seen in South Africa, oftentimes a high number of cases lie in border areas (Hiwat et al. 2018).

One of the main reasons for the increased migration from these countries into South Africa includes the search for a better livelihood in South Africa, and it has been proven that human migration plays an important role in malaria importation. A meta-analysis study on migration by Ahmed et al. (2020) showed that people with a travel history are fourteen times more likely to have malaria infection than those with no history of travel, and the study also noted that there is a tendency for malaria importation from rural areas to urban and sub-urban areas.

Various studies have reported on the capability of ITN and IRS to protect against as well as reduce malaria transmission (Okumu and Moore 2011; Pluess et al. 2010). In fact, they form the mainstay of malaria control in many countries (Tangena et al. 2020; Fernández Montoya et al. 2022). With only 0.1% of the tested people having access to both IRS and malaria protective measures respectively call for concern. The long-term implication of this is that the persistence of imported cases may cause a re-establishment of the disease in locations where it is already controlled. In this regard, the modalities of communicating health education message and coverage of indoor residual spray (IRS) need to be re-evaluated with the aim of identifying possible gaps and improving on it.

In the same vein, it is worthy of note that these high incidences of imported cases can have attendant negative impacts in the form of straining the financial system and over-stretching the available manpower as well as health resources in the province. Furthermore, there is a possibility of the introduction of new strains of malaria parasites strains to the South African populace which can lead to genetic mutation with attendant drug resistance such as reported in the Greater Mekong Sub-region and East Africa (Zhu et al. 2022).

As a means of checking the cross-border malaria challenges, the Southern African countries have put in place the “Elimination-8” body which comprises government representation from Angola, Botswana, Eswatini, Mozambique, Namibia, South Africa, Zambia, and Zimbabwe. Also, a mini-regional alliance exists between Mozambique, Eswatini, and South Africa in the form of the “MOSAWA Cross Border Initiative” with the central goal of collaborating to limit the spread and ultimately eliminate malaria among partner countries (Elimination-8 annual report, 2022; MESA 2018). Although having collaborative bodies with the aim of ending malaria in the region is a laudable initiative, there is a need for concise practical implementation of the set objectives to achieve the set goals and targets.

Another observation from Table 1 is that over the 11 years considered, there was a high number of locally acquired infections 90.5% (51,819/57,288) among the individuals tested. This differs from the findings in a KwaZulu Natal study (another South African province sharing a border with Mozambique), where the local malaria case prevalence was reported as 2% (Raman et al. 2020). Our study showed that all the sites that recorded a consistently high number of local cases in Limpopo were located in the Vhembe district; this could be attributed to the fact that most parts of the province are rural (Ramaano 2021), and the majority of the people fall in the low socioeconomic class in a provincial with the declining gross domestic product (GDP) sitting at negative 7.2 in 2020 (Limpopo Provincial Treasury 2022). A study by Bi and Tong (2014) which evaluated the link between malaria and poverty noted that malaria and poverty shared a common ground, especially among people at the county level and individuals living at the border side of the country, and the study noted that poverty may be the driver of malaria in the identified communities.

Data analysis using the time series approach allowed for careful observation of embedded trends, the reason for the trend, and the identification of any systemic pattern. The yearly plot of the recorded cases (January 2010–December 2020) is shown in Fig. 3. It inferred that there was an increasing trend and seasonal variation, and the plotted graph showed that the data was non-stationary. The year 2010 witnessed a high incidence followed by reductions in 2011 and 2012. However, there was a sharp increase in 2013, but the peak of incidence was in 2017 (Figs. 2 and 3). This observation could be due to a lack of tenacity in program implementation from year to year or the non-implementation of acquired health knowledge on the part of the communities either as a result of a conscious grip on religious or cultural beliefs. Given the fact that the effect of climate change is being felt in many countries worldwide, documented findings have shown that Limpopo Province is not spared (Rankoana 2020). Changes in rainfall, humidity, and temperature affect malaria vector both in malaria transmission and non-transmission areas resulting in changes in the critical threshold needed for parasites (Nissan et al. 2021).

Malaria cases in January, February, March, April, and October are above the average mark of 1, while those in May, June, July, August, September, November, and December are below the average, according to the seasonal index plot for the monthly analysis of recorded cases over the 11-year period (132 months). Following adjustment of the averages, January had the highest malaria incidence while the lowest was in August in Fig. 7. A possible explanation for this is the fact that in Limpopo, rainfall in the spring (September, October, November) creates conducive breeding environments for mosquitoes resulting in high malaria incidences during the summer (Adeola et al. 2019). The spontaneous increase in outbreak noted in 2017 was reportedly due to a general increase in rainfall and humidity in most countries of southern Africa (NICD 2017).

Different orders of SARIMA were considered and tried but SARIMA (3,1,1) x (3,1,0) [12] showed better precision; hence, SARIMA (3,1,1) x (3,1,0) [12] was the model used in predicting expected malaria case incidences for the three consecutive years predicting a decline in malaria incidences for these incoming years. Nevertheless, it is necessary to add that variation to this forecast may arise due to an unforeseen disease outbreak, like that of COVID-19 in the province bearing in mind that COVID-19 which shared similar shared similar symptoms with malaria and led to many misdiagnosed cases in the recent past in the province (NICD 2022). Although various studies have used different statistical modeling methods to predict malaria case incidence in Limpopo Province (Kim et al. 2019; Abiodun et al. 2019; Sehlabana et al. 2020; Martineau et al. 2022; Gondwe et al. 2021; Liu et al. 2023), SARIMA (3,1,1) x (3,1,0) [12] is a simple prediction tool which make use of surveillance data in the form of passively and actively collected malaria data for future prediction, thus assisting stakeholders in terms of preparedness towards malaria control and elimination.

## Study limitation

This study has been done within the limits of available surveillance data; however, there are gaps identified in the field data collected. This is probably due to the hybrid methods of collection. The transition to the computer-based method will allow for uniformity and robustness of collected data. A sporadic outbreak of COVID-19 could limit the outcome of the forecasted case incidences.

## Conclusion

The analysis of this 11-year data has demonstrated that imported malaria accounted for 9.5% of all recorded malaria cases in Limpopo Province. This is a significant factor posing a challenge to the malaria elimination efforts in the province. Planning for an interventional approach in conjunction with a simple model forecast will foster an effective reduction of malaria case incidences in the province. Although there are malaria control measures that are already in place, based on the study findings, there is a need to strongly consider the strengthening of health education campaigns on malaria prevention methods at the grass-root level as well as the strengthening of indoor residual spray programs. The collaborating countries in the Southern Africa region need to re-evaluate the objectives of the alliance and consider practical steps to achieving them in order to reduce imported malaria in participating countries.

## Data Availability

The data that support the findings of this study are available from the Office of Malaria Control Program, Limpopo Department of Health, South Africa but restrictions apply to the availability of these data, which were used under license for the current study, and so are not publicly available. Data are however available from the authors upon reasonable request and with permission of the Office of Malaria Control Program, Limpopo Department of Health, South Africa.
